# Supplementary material for: Comparative RNA-Seq Analysis Revealed Tissue-Specific Splicing Variations during the Generation of the PDX Model
Source: Int J Mol Sci. 2023 Nov 30;24(23):17001. doi: 10.3390/ijms242317001 (PMC10707456; doi:10.3390/ijms242317001)
Supplement: Supplementary file 1 [file ijms-24-17001-s001.zip › ijms-2719418-supplementary.pdf]

Supplementary Figure Legends

Supplementary Figure S1. Pictures of PDX mouse and tumors in four types of tumor

A-D. Pictures of PDX mouse (upper panels) and dissected tumors (lower panels) of gastric (KY009) (A), lung (KY011) (B), breast (KY013) (C) and colon (KY018) (D).

Supplementary Figure S2. RNA biotype distribution of the four primary-PDX pairs shows conserved pattern in each tumor type

A-D. RNA biotype distribution of gastric (A), lung (B), breast (C), colon (D). Grey bar indicate genome portion and hatched bar shows detected level. Solid bar shows percent in sample. Left panel is for primary tumor and right panel is for PDX. Note that primary-PDX pair shows quite similar RNA type distribution with a slight difference in gastric, where PDX RNA contains more antisense RNAs.

Supplementary Figure S3. DEG analysis for four primary-PDX pairs reveals distinct pattern of gene expression changes

A. Heatmap of differentially expressed genes for each primary-PDX pair. Upper row shows primary tumor while lower row is for PDX tumor. Red color indicates upregulation and blur is for downregulation of the expression.
B. Venn-diagram presenting the number of differentially expressed genes, overlapped with tissue-specific gene lists from two publicly available databases, TIGER (red circle) and TissGDB (orange circle).

Supplementary Figure S4. Up-regulated pathway analysis for three primary-PDX pairs

A-C. Gene ontology (upper panel) and KEGG pathway results (lower panel) for gastric (A), lung (B) and breast cancer (C). The enriched pathways are listed on Y axis and X-axis marks gene ratio. Size of dots indicate the number of genes included in the pathway. Color of dots represent statistical significance. BP; Biological Process, CC; Cellular Compartment, MF; Molecular Function

Supplementary Figure S5. Distribution of differential splicing events in four primary-PDX pairs reveal frequent exon skipping during PDX formation

A-D. Number of differential splicing events with high (FDR<0.05, in pink) and low significance (FDR>0.05, in blue) in gastric (A), lung (B), breast (C) and colon (D) tumors. SE; Skipped Exon. E-H. Percent of high (FDR<0.05) or low (FDR>0.05) confidence splicing variations in four different tumor types.

Supplementary Figure S6. Molecular analysis of differential splicing events during PDX formation in four tumor types

A. Graph presenting average difference of the splicing events during the PDX formation of four tumor types. B. Agarose gel picture of the RT-PCR product for the CASK gene in breast tumor (KY013), showing multiple bands increased in PDX sample. C. RT-PCR results for the splicing products of CD44 gene in lung tumor (KY011), showing unspliced transcript. RPL13a was used as a internal control

Supplementary Figure S1

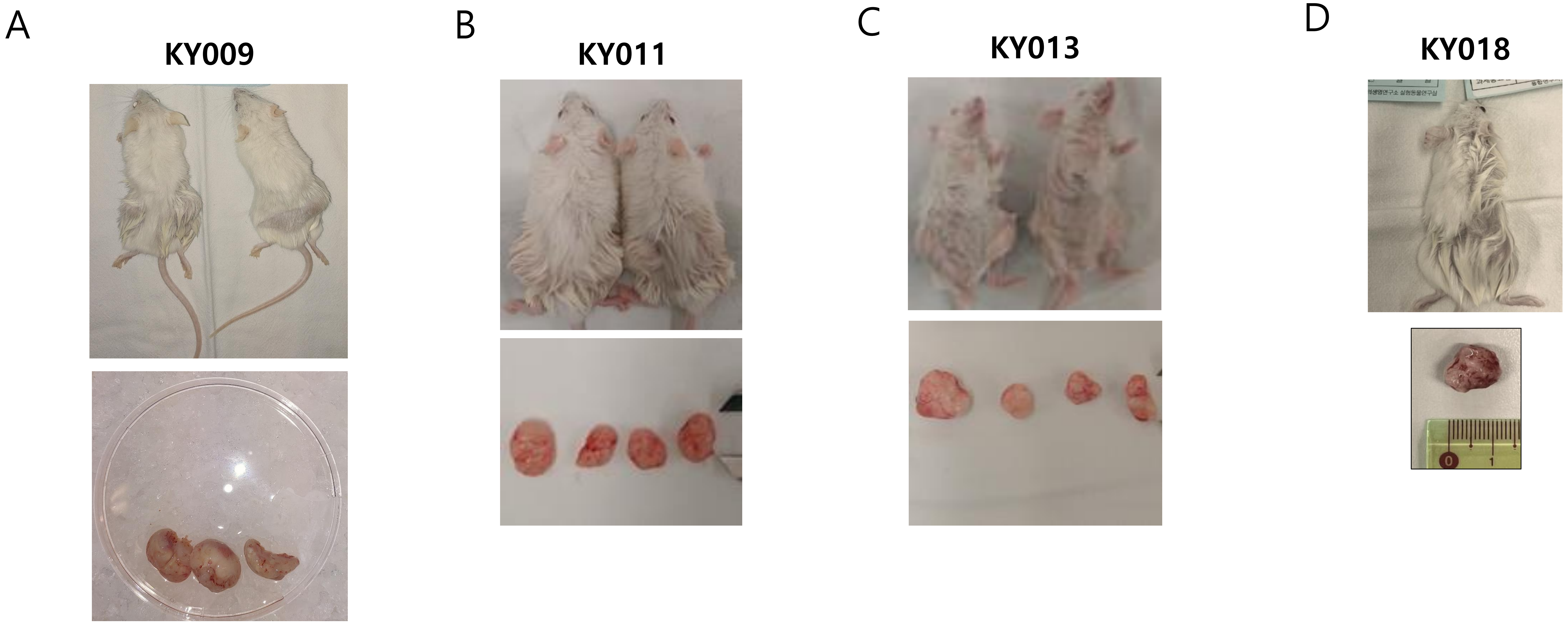

Supplementary Figure S2

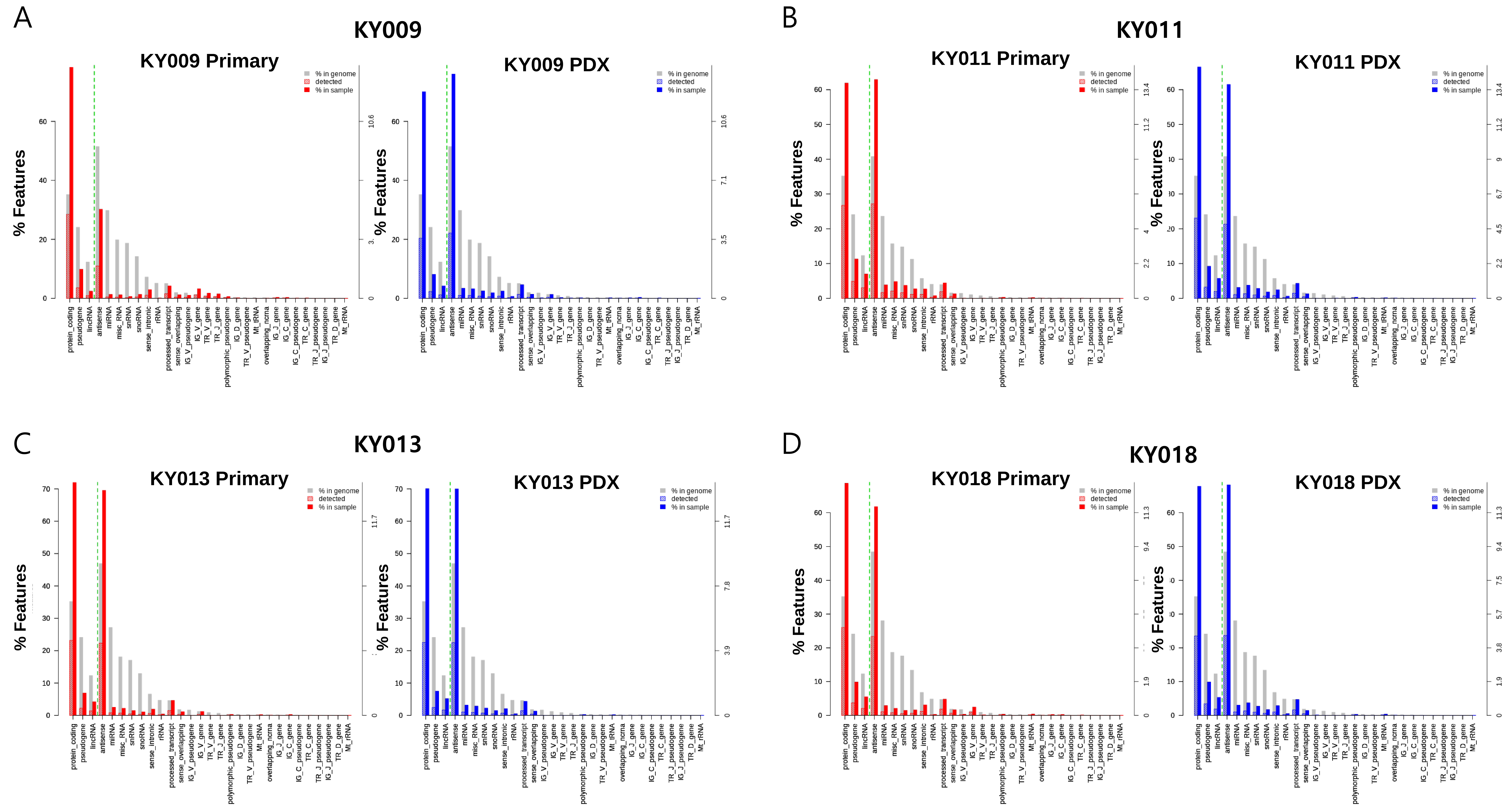

Supplementary Figure S3

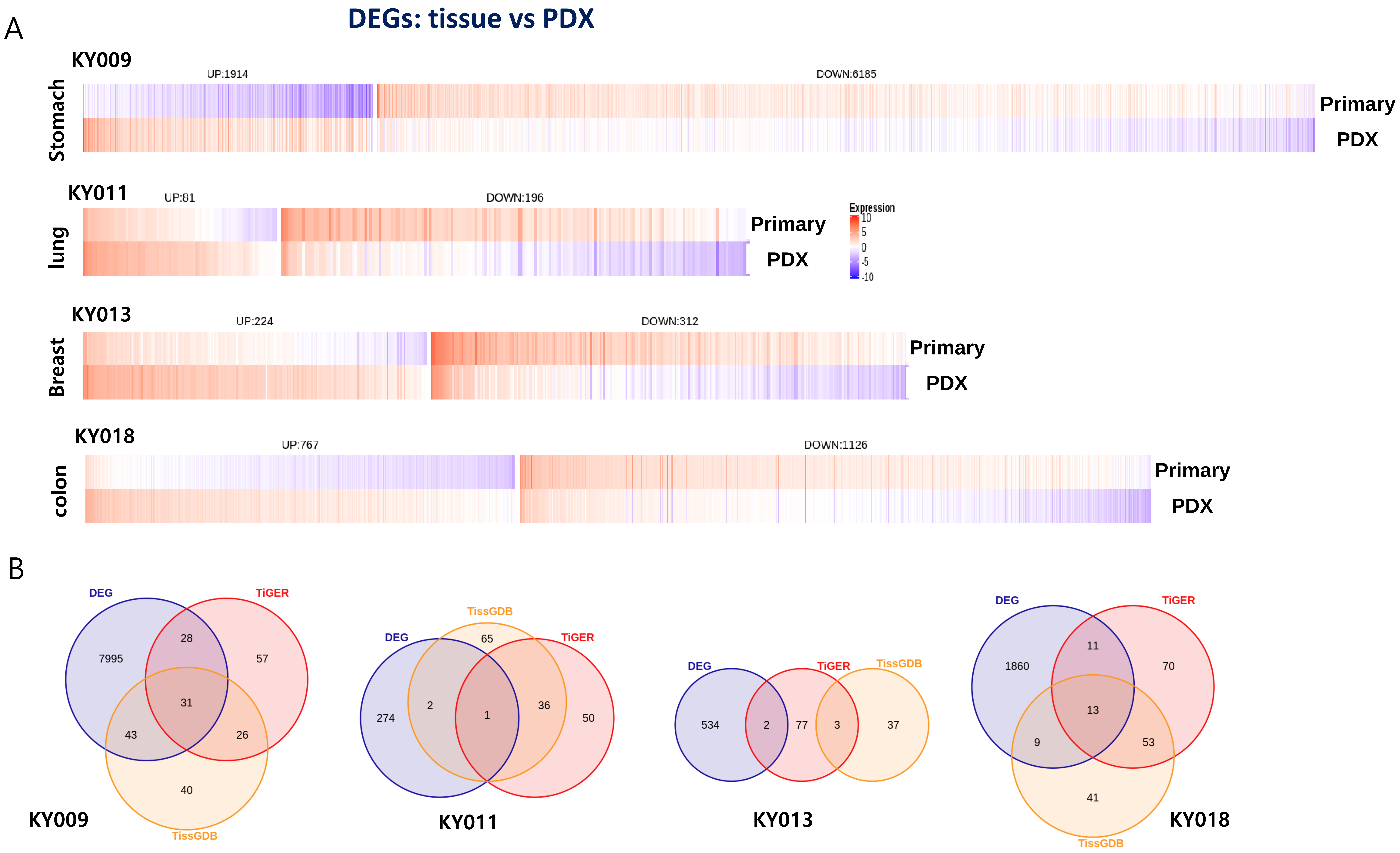

Supplementary Figure S4

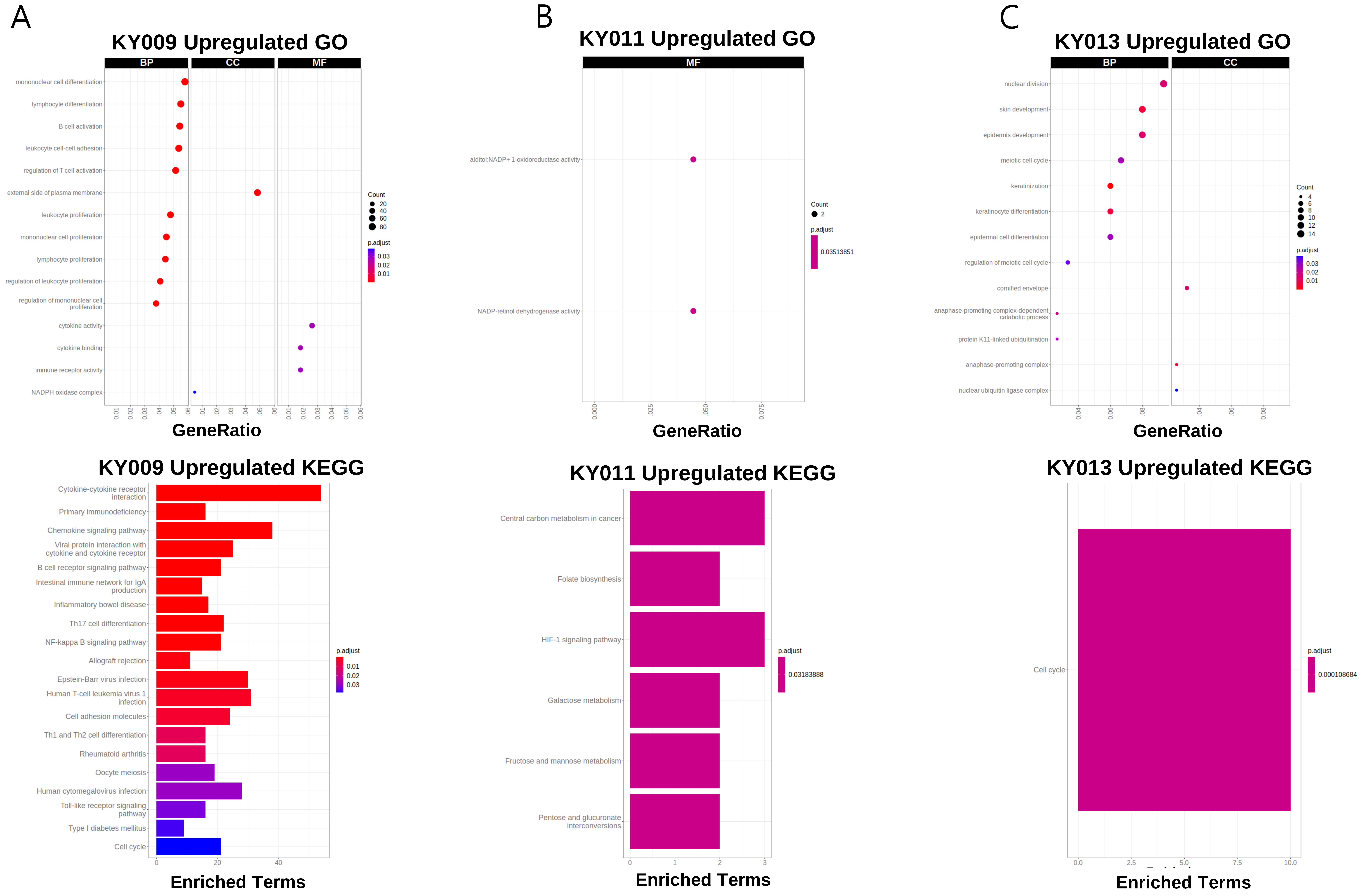

Supplementary Figure S5

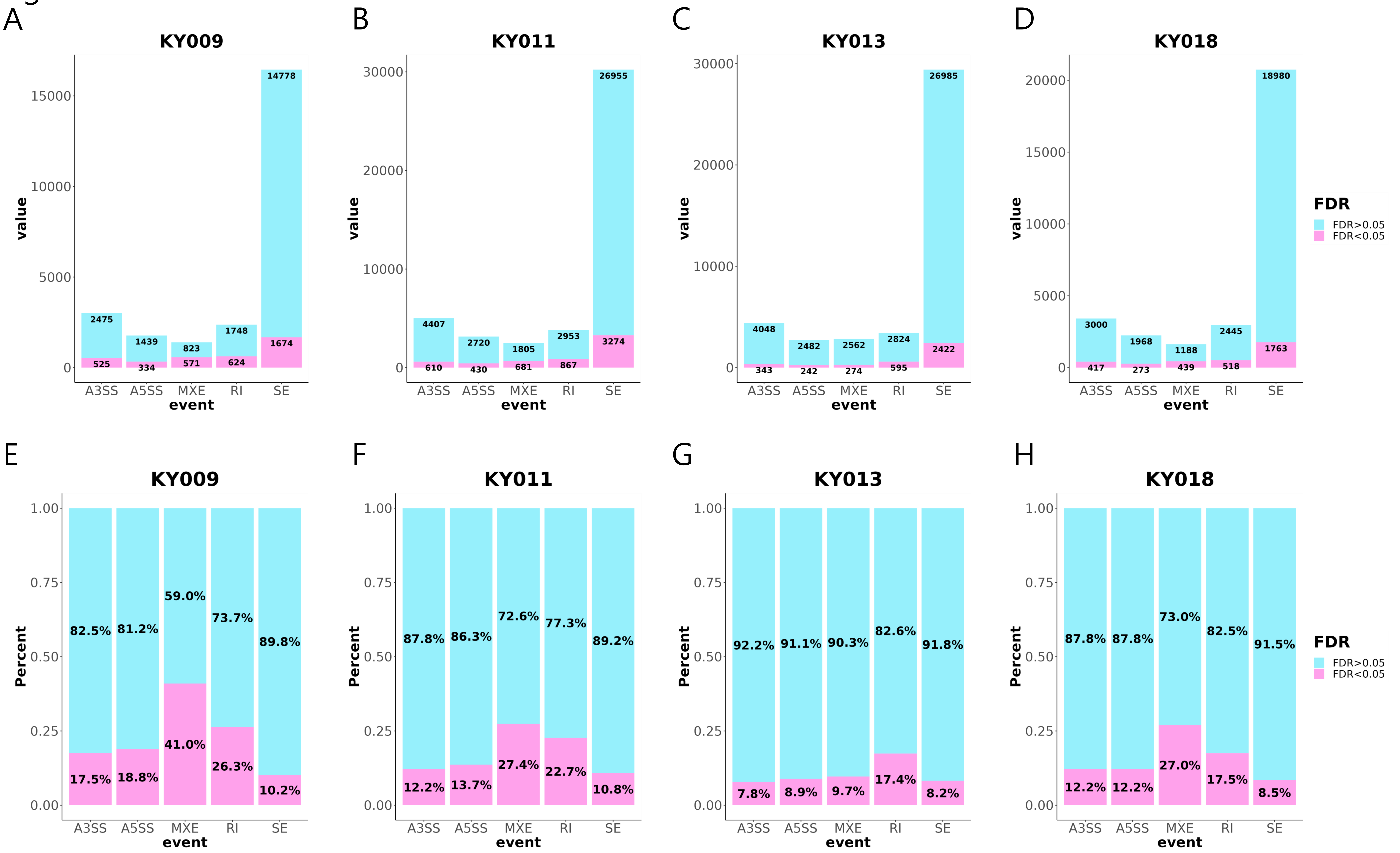

Supplementary Figure S6

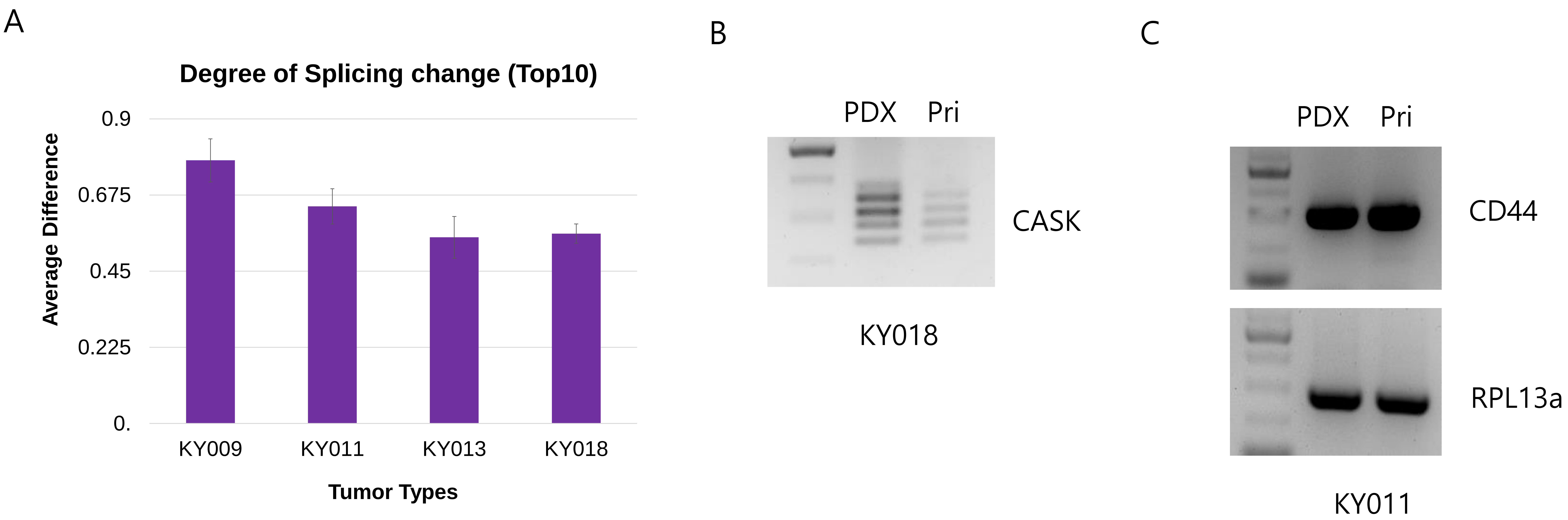

Supplementary Table S1. Information of PDX tumor analyzed in this study

ID	Gen/Age	Cancer Type	PDX Graft Date	PDX harvest Date
KY_009	M/63	Stomach	2020-12-01	2020-12-28
KY_011	M/72	Lung	2021-01-15	2021-03-16
KY_013	F/49	Breast	2021-02-19	2021-03-29
KY_018	F/79	Colon	2022-08-05	2022-09-25

Supplementary Table S2. Top 10 differentially expressed genes in stomach primary tumor/PDX pair

Gene_id	Gene_ Symbol	KY009_ TUMOR	KY009_ PDX	TUMOR_ RPKM	PDX_ RPKM	Log2FC	Difference	Up/Down in PDX
ENSG00000152348.11	ATG10	221	6408	1.28	1586.57	10.28	1585.29	UP
ENSG00000167476.6	JSRP1	36	2225	0.58	1535.46	11.37	1534.88	UP
ENSG00000143882.5	ATP6V1C2	7	3121	0.07	1266.74	14.22	1266.67	UP
ENSG00000157734.9	SNX22	25	4360	0.14	1019.98	12.87	1019.84	UP
ENSG00000109686.12	SH3D19	9787	6105	39.62	1057.45	4.74	1017.83	UP
ENSG00000096088.12	PGC	127356	1	1983.10	0.67	-11.54	1982.43	DOWN
ENSG00000160182.2	TFF1	35065	0	2006.65	1.22	-10.68	2005.43	DOWN
ENSG00000215182.6	MUC5AC	275134	0	1462.98	0.11	-13.65	1462.87	DOWN
ENSG00000160181.4	TFF2	33547	0	1274.46	0.81	-10.61	1273.65	DOWN
ENSG00000169605.5	GKN1	43324	0	1188.94	0.59	-10.98	1188.35	DOWN

Supplementary Table S3. Top 10 differentially expressed genes in lung primary tumor/PDX pair

Gene_id	Gene_ Symbol	KY011_ TUMOR	KY011_ PDX	TUMOR_ RPKM	PDX_ RPKM	Log2FC	Difference	Up/Down in PDX
ENSG00000170477.8	KRT4	33	134	4.42	45.14	3.35	40.72	UP
ENSG00000105388.10	CEACAM5	26	93	3.44	30.96	3.17	27.52	UP
ENSG00000086548.8	CEACAM6	59	134	8.58	48.99	2.51	40.41	UP
ENSG00000120093.7	HOXB3	141	321	8.39	48.03	2.52	39.64	UP
ENSG00000165092.8	ALDH1A1	151	243	23.52	95.16	2.02	71.64	UP
ENSG00000204540.6	PSORS1C1	9322	43	715.66	8.30	-6.43	707.36	DOWN
ENSG00000167768.4	KRT1	784	3	139.85	1.35	-6.70	138.51	DOWN
ENSG00000047315.10	POLR2B	1763	35	132.59	6.62	-4.32	125.98	DOWN
ENSG00000215375.2	MYL5	954	14	98.35	3.63	-4.76	94.72	DOWN
ENSG00000168542.8	COL3A1	907	0	59.06	0.08	-9.50	58.98	DOWN

Supplementary Table S4. Top 10 differentially expressed genes in breast primary tumor/PDX pair

Gene_id	Gene_ Symbol	KY013_T UMOR	KY013_ PDX	TUMOR_ RPKM	PDX_ RPKM	Log2FC	Difference	Up/Down in PDX
ENSG00000116745.6	RPE65	2	131	0.837633	52.42	5.97	51.58	UP
ENSG00000215067.5	NA	62	402	21.06711	130.51	2.63	109.44	UP
ENSG00000146648.11	EGFR	59	532	4.975971	42.87	3.11	37.89	UP
ENSG00000203684.5	NA	2	23	3.410643	37.47	3.46	34.06	UP
ENSG00000130822.11	PNCK	64	399	13.74172	81.85	2.57	68.11	UP
ENSG00000198865.5	CCDC152	2567	140	817.8422	42.62	-4.26	775.23	DOWN
ENSG00000049246.10	PER3	2122	9	287.825	1.17	-7.95	286.66	DOWN
ENSG00000267954.1	NA	242	22	354.1271	30.76	-3.53	323.37	DOWN
ENSG00000184330.7	S100A7A	599	27	146.909	6.33	-4.54	140.58	DOWN
ENSG00000256040.1	PAPPA-AS1	238	9	106.3181	3.84	-4.79	102.48	DOWN

Supplementary Table S5. Top 10 differentially expressed genes in colon primary tumor/PDX pair

Gene_id	Gene_ Symbol	KY018_ TUMOR	KY018_ PDX	PDX_ RPKM	TUMOR_RP KM	Log2FC	Difference	Up/Down in PDX
ENSG00000156928.4	MALSU1	59	720	131.77	7.20	4.19	124.57	UP
ENSG00000154640.10	BTG3	79	429	131.11	16.10	3.03	115.02	UP
ENSG00000103061.7	SLC7A6OS	250	1113	151.23	22.65	2.74	128.58	UP
ENSG00000153551.9	CMTM7	74	367	68.96	9.27	2.90	59.69	UP
ENSG00000070731.5	ST6GALNAC2	625	2350	219.13	38.86	2.50	180.28	UP
ENSG00000007306.10	CEACAM7	1417	29	7.72	251.35	-5.03	243.63	DOWN
ENSG00000148346.7	LCN2	710	8	2.74	162.19	-5.89	159.45	DOWN
ENSG00000132464.7	ENAM	1990	0	0.05	143.37	-11.37	143.32	DOWN
ENSG00000102837.6	OLFM4	904	35	8.51	146.63	-4.11	138.11	DOWN
ENSG00000086548.8	CEACAM6	3594	281	64.89	553.33	-3.09	488.44	DOWN

Supplementary Table S6. Top 10 differentially spliced genes in stomach primary tumor/PDX pair

ID	gene Symbol	chr	strand	exonStart _base	exonEnd	FDR	IncLevel1	IncLevel2	IncLevel Difference
40699	CTNND1	chr11	+	57583386	57583473	0	0.961	0.072	0.889
26633	SLK	chr10	+	105770573	105770666	0	0.91	0.073	0.837
42948	LMO7	chr13	+	76383289	76383319	0	0.032	0.866	-0.834
11986	MPZL1	chr1	+	167745300	167745403	0	0.944	0.121	0.823
30011	PUM2	chr2	-	20478343	20478580	0	0.959	0.18	0.779
543	ACOT9	chrX	-	23752457	23752484	1.51E-11	0.058	0.827	-0.769
13200	SEC31A	chr4	-	83752089	83752128	1.04E-12	0.848	0.111	0.737
21634	RABGAP1L	chr1	+	174846529	174846743	4.60E-11	0.956	0.244	0.712
40693	CTNND1	chr11	+	57558856	57559145	4.44E-12	0.155	0.854	-0.699
14912	FLNB	chr3	+	58127584	58127623	4.21E-10	0.891	0.198	0.693

Supplementary Table S7. Top 10 differentially spliced genes in lung primary tumor/PDX pair

ID	gene Symbol	chr	strand	exonStart _0base	exonEnd	FDR	IncLevel1	IncLevel2	IncLevel Difference
36880	CD44	chr11	+	35219667	35219793	0	0.795	0.052	0.743
36903	CD44	chr11	+	35229651	35229753	0	0.72	0.029	0.691
21625	RCOR3	chr1	+	211486061	211486177	0	0.78	0.106	0.674
23686	POLR2J3	chr7	-	102207028	102207183	0	0.806	0.136	0.67
19713	MYL6	chr12	+	56553370	56553406	0	0.978	0.334	0.644
43726	IFT20	chr17	-	26659171	26659207	0	0.773	0.143	0.63
5857	COASY	chr17	+	40714313	40714505	1.35E-08	0.847	0.226	0.621
37731	PPHLN1	chr12	+	42726461	42726504	2.80E-09	0.93	0.329	0.601
5858	COASY	chr17	+	40714373	40714505	4.84E-06	0.779	0.204	0.575
9507	REPIN1	chr7	+	150067848	150067973	4.68E-09	0.822	0.256	0.566

Supplementary Table S8. Top 10 differentially spliced genes in breast primary tumor/PDX pair

ID	gene Symbol	chr	strand	exonStart _0base	exonEnd	FDR	IncLevel1	IncLevel2	IncLevel Difference
24774	RABGAP1L	chr1	+	174927027	174927310	9.06E-09	0.852	0.17	0.682
2492	RBCK1	chr20	+	401514	401675	5.39E-09	1	0.394	0.606
40951	SENP6	chr6	+	76350399	76350420	2.41E-05	0.863	0.278	0.585
504	CASK	chrX	-	41416284	41416353	0.0001725	0.87	0.313	0.557
20634	RHOC	chr1	-	113247721	113247790	1.98E-06	0.684	0.166	0.518
5211	FAM104A	chr17	-	71208816	71208879	8.90E-06	0.623	0.105	0.518
3684	OCEL1	chr19	+	17339611	17339724	0.0005821	0.377	0.886	-0.509
28940	PLD3	chr19	+	40871624	40871837	2.83E-05	0.881	0.378	0.503
23937	SERPINA1	chr14	-	94854896	94854997	3.64E-10	0.351	0.843	-0.492
503	CASK	chrX	-	41414852	41414888	0.0018157	0.858	0.391	0.467

Supplementary Table S9. Top 10 differentially spliced genes in colon primary tumor/PDX pair

ID	gene Symb ol	chr	strand	exonStart _0base	exonEnd	FDR	IncLevel1	IncLevel2	IncLevel Difference
28152	FAM45A	chr10	+	120864275	120864534	7.27E-11	0.909	0.314	0.595
28989	DNAJB6	chr7	+	157202488	157208223	0.0035434	0.407	1	-0.593
29393	APOPT1	chr14	+	104040443	104040507	6.42E-06	0.141	0.724	-0.583
21329	SERPINA1	chr14	-	94854896	94855000	2.30E-13	0.285	0.861	-0.576
21327	SERPINA1	chr14	-	94854896	94854997	5.76E-13	0.306	0.862	-0.556
28594	INSIG2	chr2	+	118853994	118854376	9.06E-07	0.397	0.932	-0.535
32248	TPM1	chr15	+	63336225	63336351	4.60E-05	0.476	1	-0.524
24649	C1orf116	chr1	-	207200838	207201024	1.14E-05	0.151	0.658	-0.507
37901	ALG11	chr13	+	52598141	52599073	4.24E-05	0.389	0.893	-0.504
19028	CCDC58	chr3	-	122090521	122090647	6.65E-08	0.384	0.88	-0.496

Supplementary Table S10. Primers used in this study

표 1

Gene	Forward	Reverse
CTNND1	CTCTCTCCTTCCTGCTTCC	TGGGGTCCGTTGAGAGTTTCC
CD44	CAGCCTCAGCTCATACCAGC	TGCTTGATGTCAGAGTAGAAG
CASK	GCGGGGGAGTATTACCTTC	GAAGGAATGAGACCTGCAG
SERPINA1	GGCGGCAGTAAGTCTTCAGC	TGGGTGATCCTGATCATGG
EPB41L2	CATTGCCGTGGTACAAGATG	TTCGTCTTTCCCAACCTCTG
FBLN2	CTCCTGTTTTCCCGGCTATG	TGCGTGCCTTGTAGCAGTGG
SLK1	CAGAATATGCTGAAGAACCG	CTCTCATGAGCTGTTGCTTG
COASY	GTGGAACCCAAGCCTTGAGG	GTGTGTGATTCACCAGCCGG
SENP6	GTCATTATGTTCTGGAACTG	ACCTTGGAGCCGACTTAACC
AOPOT1	AGACAAGAAACACAAGAATGG	GTTATAATACATGTGCTTCTG
